# Basal Forebrain Impairment: Understanding the Mnemonic Function of the Septal Region Translates in Therapeutic Advances

**DOI:** 10.3389/fncir.2022.916499

**Published:** 2022-05-31

**Authors:** Marian Tsanov

**Affiliations:** UCD School of Medicine, University College Dublin, Dublin, Ireland

**Keywords:** medial septum, basal forebrain amnesia, Alzheimer’s disease, Lewy body dementia, attention, reward, locomotion, dopamine

## Abstract

The basal forebrain is one of the three major brain circuits involved in episodic memory formation together with the hippocampus and the diencephalon. The dysfunction of each of these regions is known to cause anterograde amnesia. While the hippocampal pyramidal neurons are known to encode episodic information and the diencephalic structures are known to provide idiothetic information, the contribution of the basal forebrain to memory formation has been exclusively associated with septo-hippocampal cholinergic signaling. Research data from the last decade broadened our understanding about the role of septal region in memory formation. Animal studies revealed that septal neurons process locomotor, rewarding and attentional stimuli. The integration of these signals results in a systems model for the mnemonic function of the medial septum that could guide new therapeutic strategies for basal forebrain impairment (BFI). BFI includes the disorders characterized with basal forebrain amnesia and neurodegenerative disorders that affect the basal forebrain. Here, we demonstrate how the updated model of septal mnemonic function can lead to innovative translational treatment approaches that include pharmacological, instrumental and behavioral techniques.

## Introduction

Hippocampus, limbic diencephalon and basal forebrain are established as the three major foci of memory formation (Penfield and Milner, 1958; Victor et al., 1971; Damasio et al., 1985). The basal forebrain is a heterogenous region, composed of several areas: substantia innominate, nucleus accumbens and ventral pallidum of the basal ganglia, bed nucleus of the stria terminalis, the preoptic area, the nucleus basalis of Meynert, diagonal band of Broca and the septal nuclei. Memory impairment due to basal forebrain dysfunction is often characterized with multiple lesioned areas rather than a single region of the basal forebrain (Alexander and Freedman, 1984; Vilkki, 1985; Parkin et al., 1988). As a result, it has been difficult to pinpoint the exact anatomical correlate of basal forebrain amnestic syndrome. Sufficient evidence from the last two decades indicates that the medial septum mediates mnemonic function of the basal forebrain. Here, we will first examine the type of memory impairment in basal forebrain amnesia. We will then examine the specific contribution of human medial septum to this type of memory impairment. Next, we will review the animal studies that investigate what type of information is processed by the medial septum. Finally, we will propose how the laboratory-based findings could translate in efficient therapeutic strategies for patients with basal forebrain impairment (BFI).

## Basal Forebrain Amnesia

The clinical research about the role of the basal forebrain in memory formation is based to a large degree on findings about disrupted circulation of the anterior communicating artery (AComA). Impaired circulation of AcomA causes basal forebrain amnesia. More than 80 years ago AComA hemorrhage was associated with amnesia (Lindqvist and Norlen, 1966; Talland et al., 1967). Since then, AComA aneurysm rupture has been consistently linked to severe memory impairment (Deluca and Locker, 1996; Johnson et al., 1997; Beeckmans et al., 1998; Bottger et al., 1998; Simard et al., 2003; Martinaud et al., 2009; Nassiri et al., 2018; Ma et al., 2021). AComA is supplying the basal forebrain with blood and the symptoms of AComA aneurysm rupture were linked to basal forebrain lesions (Damasio et al., 1985; Irle et al., 1992; Morris et al., 1992). What type of memory impairment is caused by AComA hemorrhage? To answer this question, we need to review the seminal clinical reports. Anterograde amnesia, which is the inability to form new memories, was observed in 10 patients who suffered aneurysm rupture and AComA surgery (D’esposito et al., 1996). In some cases, the study reported temporally graded retrograde amnesia, indicating impaired memory retention. AComA aneurysm in another 10 patients evoked amnestic pattern of impaired acquisition but spared reversal (Myers et al., 2006). Ruptured and repaired AComA in 10 patients caused dysfunctional memory with impaired recall (Diamond et al., 1997). The authors indicated that there was no impairment of the limbic cortices, and the cause of the amnesia was linked to the basal forebrain structures. Deficits in the attention, short- and long-term memory were observed in 28 patients after AComA surgery (Hutter and Gilsbach, 1996). Rupture of AComA and AComA surgery in 18 patients resulted in impairment of the explicit memory with concurrent preservation of the implicit memory. Explicit memory is long-term human memory that involves episodic and semantic features (see Box 1). Ruptured aneurysm of AComA in 59 patients induced retrieval difficulties in episodic and semantic memory tasks (Simard et al., 2003). Episodic autobiographical memory impairment was identified in five patients with damage to the basal forebrain due to AComA aneurysm rupture or resection of an arteriovenous malformation (Damasio et al., 1985). Episodic memory comprises personal experience about time, place and context (Tulving, 2002; Addis et al., 2004). These studies show that the patients with AComA aneurysm where the lesions have been localized to the basal forebrain, share similar memory impairments that can be defined as basal forebrain amnestic syndrome (Phillips et al., 1987; Morris et al., 1992; Abe et al., 1998). In summary, basal forebrain amnesia impairs anterograde memory, explicit episodic and semantic memory, short- and long-term memory (see Box 1). All these features indicate hippocampus-dependent memory impairment and pinpoint the septo-hippocampal circuitry as the likely focus of dysfunction. The contribution of medial septum to memory has been confirmed in animal studies. Lesions of the medial septum resulted in pronounced memory deficits for hippocampus-dependent spatial tasks, including the Morris water maze task and radial maze task (Kelsey and Landry, 1988; M’harzi and Jarrard, 1992; Rashidy-Pour et al., 1996). The findings from the septal lesions suggested that septo-hippocampal pathways contribute to system consolidation of a spatial memory (Lecourtier et al., 2011; Okada et al., 2015). The improvement of the imaging resolution together with the accumulation of cases provided us with the perfect opportunity to validate the mnemonic role of human medial septum.

**Box 1. Types of memory.**

**Long-term** memory: storage of information for an extended period of time. There is no unified view of how much time is required for memory to be defined as long-term but it is generally accepted that memory lasting more than 24 h is a long-term memory

**Implicit** (non-declarative) memory: long-term memory for skills, habits, and conditioned responses

**Explicit** (declarative) memory: long-term memory for facts and events

**Semantic** memory: explicit memory for factual information

**Episodic** memory: explicit memory for personal experiences

**Autobiographical** memory: semantic and episodic memory for personal history

**Spatial** memory: episodic memory for allocentric-based orientation and navigation

**Anterograde** amnesia: inability to form new memories after the event that caused the amnesia

**Retrograde** amnesia: inability to recollect memories formed prior the event that caused the amnesia

## Mnemonic Medial Septum

AComA hemorrhage could affect multiple regions of the basal forebrain as well as neighboring structures such as the orbitofrontal and medial frontal cortices and the caudate nucleus. Because of its small size in humans, the clinical findings about the dysfunction of the medial septum for a long time have been blended with the other nuclei of the basal forebrain. The septal contribution to memory was initially proposed in studies with basal forebrain amnesia where the septal region was damaged and nucleus basalis was spared (Phillips et al., 1987; Morris et al., 1992; Goldenberg et al., 1999). The early data about the mnemonic role of the septal region involves case studies of single patients. A patient presented with anterograde amnesia following rupture and repair of an arteriovenous malformation in the atrium of the left ventricle (Von Cramon and Schuri, 1992). The authors explained these findings with impairment of the septal region, the anterior and posterior singular gyrus/cingulate bundle and the fornix (Von Cramon and Schuri, 1992). Another patient with bilateral septal damage of the basal forebrain including the septal region became deficient in long-term memory (Von Cramon et al., 1993). It was proposed that the septal area was part of larger memory and learning processing network (Von Cramon et al., 1993). These findings supported the proposal that damage to the medial septum and diagonal band complex was most likely the primary cause for impaired memory in basal forebrain-damaged patients due to a dysfunctional AcomA. Some of the studies investigated also the type of memory impairment after focal septal lesions. A postoperative magnetic resonance imaging (MRI) study reported a patient who developed primarily anterograde amnesia after clipping of an unruptured anterior communicating artery aneurysm (Abe et al., 1998). The patient also developed mild retrograde amnesia for both semantic and personal episodic information. This was caused by a discrete lesion, centered in the right diagonal band of Broca and the septal nucleus. Concurrently, the nucleus basalis of Meynert was minimally affected and the diencephalon was not damaged. The authors concluded that disconnection of the pathway between the diagonal band of Broca and the hippocampus contributed to declarative memory impairment (Abe et al., 1998). A patient with a rare septum pellucidum cyst presented with memory impairment, an MRI scan showed that the cyst was limited to the septal region (Chen et al., 2017). Mini-Mental State Exam (MMSE) and Montreal Cognitive Assessment (MoCA) revealed mild cognitive impairment before the treatment of the cyst. After neuroendoscopic fenestration of the cyst, the patient’s memory has improved. The authors concluded that the patient’s episodic memory disorder was due to a secondary septal lesion attributed to the cyst (Chen et al., 2017). Studies in healthy participants extended the focus of septal contribution to memory formation. Medial septum and diagonal band of Broca were investigated in the context of proactive interference paradigm (i.e., previous learning impairs the acquisition of new, related information). Analyses of fMRI from human participants showed that septal activity covaried with activity in areas important to selective attention and memory (De Rosa et al., 2004; Caplan et al., 2007). These findings supported the concept of septum-dependent functional network involved in episodic memory (Caplan et al., 2007). To identify the link between the septal nuclei and memory formation a study used high-resolution magnetic resonance imaging scans from healthy participants and the septal forebrain volume was calculated using probabilistic cytoarchitectonic maps (Butler et al., 2012). The data showed that bilateral septal forebrain volume was a significant positive predictor of recognition memory accuracy. Larger septal forebrain volume was associated with the ability to recall item source/context accuracy (Butler et al., 2012). Together this line of research validated the fundamental role of medial septum for the declarative episodic memory and established the contribution of septal dysfunction to anterograde amnesia. Laboratory-based fundamental findings elucidated one of the mechanisms by which medial septum supports memory formation. Neurons in the medial septum fire in bursts phase-locked in theta frequency within the hippocampus and entorhinal cortex (Stewart and Fox, 1989, 1990). Their rhythmic pace-maker activity is involved in the generation of theta rhythm across the limbic circuitry and medial temporal lobe (Petsche et al., 1962; Stumpf et al., 1962; Buzsáki, 2002). Theta synchronization of these structures mediates memory formation by regulating the synaptic strength between neurons. Synchronized membrane depolarizations of populations of pre- and postsynaptic cells result in enhanced temporal synaptic interaction between these neurons (Gray, 1994; Hasselmo et al., 2002; Axmacher et al., 2006). Disconnection of septo-hippocampal projections via fimbria-fornix lesions is sufficient to suppress theta in the hippocampus (Gray, 1971; Andersen et al., 1979; Sainsbury and Bland, 1981; Oddie and Bland, 1998). Focal lesion of the medial septum eliminates not only hippocampal theta oscillations but also abolishes spatial memory formation (M’harzi and Jarrard, 1992). The medial septum does not merely generate theta rhythm, but actively synchronizes hippocampal activity and sensorimotor inputs through theta cycle (Tsanov et al., 2014). There is another key mechanism by which the medial septum contributes to the formation of hippocampus-dependent memory. This is the cholinergic neuromodulatory control of medial septum on the hippocampal function. Successful treatment of patients with impaired basal forebrain should address the septal cholinergic deficits. We next examine the contribution of the cholinergic neuronal loss to BFI.

## Medial Septum is the Cholinergic Locus of Episodic Memory

A seminal case study with basal forebrain amnesia induced by localized glioma proposed that memory impairment may result from the forebrain lesion that deprived the hippocampus of the cholinergic innervation (Morris et al., 1992). Since then, the basal forebrain amnestic syndrome has been primarily associated with dysfunctional cholinergic projections from the diagonal band of Broca and the medial septum. The cholinergic neurons in the basal forebrain are divided into four groups depending on their cell body location and projections. The human-based studies adopted the nomenclature Ch1, Ch2, Ch3, and Ch4 for medial septal nucleus, vertical limb of the diagonal band, horizontal limb of the diagonal band and nucleus basalis of Meynert, respectively (Mesulam et al., 1983). The cholinergic projections form the medial septum (Ch1) and vertical limb of the diagonal band (Ch2) provides input to the limbic cortices, including hippocampus and entorhinal cortex, while the nucleus basalis of Meynert (Ch4) supplies the prefrontal cortex. Thanks to neuroimaging advances in the last 10 years, it is now possible to measure atrophy in basal forebrain Ch1-4 areas using MRI and PET and to correlate it to the clinical symptoms (Kilimann et al., 2014). Loss of cholinergic neurons in the basal forebrain is a pathoanatomical feature of some neurodegenerative disorders such as Alzheimer’s disease (AD) (Wilcock et al., 1988; Sasaki et al., 1995; Mufson et al., 2003, 2007; Mesulam, 2004, 2012; Schliebs and Arendt, 2006; Kerbler et al., 2015; Fernandez-Cabello et al., 2020) and Lewy body dementia (LBD) (Whitehouse et al., 1983; Arendt et al., 1995; Lippa et al., 1999; Tiraboschi et al., 2002; Collerton et al., 2003; Mckeith et al., 2005; Ferman et al., 2006; Klein et al., 2010; Grothe et al., 2014). LBD is an umbrella term for Parkinson’s disease (PD) dementia and dementia with Lewy bodies (DLB); the difference between them depends on whether dementia occurs in the context of an established movement disorder or prior to the parkinsonian motor symptoms (Aarsland et al., 2009; Mckeith et al., 2017). The cognitive decline in these neurodegenerative diseases is linked to disrupted cholinergic innervation from nucleus basalis to the frontal lobe, while the memory impairment is associated with dysfunctional cholinergic innervation of hippocampus (Gaspar and Gray, 1984; Iraizoz et al., 1999; Pappas et al., 2000; Schliebs and Arendt, 2006). PDD and DLB are characterized by the neuropathological hallmark of cortical Lewy bodies composed of alpha-synuclein (Hurtig et al., 2000; Horvath et al., 2013), and by marked cholinergic neurotransmitter system dysfunction (Shimada et al., 2009; Oswal et al., 2021). The cognitive decline in PDD and DLB is linked to severe degeneration of the cholinergic neurons of the basal forebrain (Lippa et al., 1999; Bohnen et al., 2003; Hilker et al., 2005; Fujishiro et al., 2006; Shimada et al., 2009; Grothe et al., 2014). The degree of cholinergic degeneration and dysfunctional cholinergic innervation correlate with the level of cognitive impairment (Iraizoz et al., 1999; Jicha et al., 2010). Volumetric measurement of the basal forebrain was shown to predict early cognitive decline (Ray et al., 2018). The authors evaluated regional basal forebrain atrophy in the early stage of PD participants alongside longitudinal evaluation of cognitive status and multi-domain cognitive assessment. This study demonstrated that basal forebrain degeneration can be detected even at very early disease stages in patients with suspected cognitive dysfunction, and can be used to predict future development of mild cognitive impairment (Ray et al., 2018). Decreased volume of the basal forebrain in patients with mild cognitive impairment correlates to the degree of episodic memory impairment (Grothe et al., 2010, 2016) and the loss of basal forebrain cholinergic projections to the hippocampus is proportional to the memory deficits observed in neurodegenerative disorders (Gargouri et al., 2019; Pereira et al., 2020). A posterior-anterior pattern of basal forebrain atrophy is proposed for the progression of PD dementia where atrophy of Ch4 is followed by changes in Ch1 and Ch2 (Pereira et al., 2020). Voxel-based whole brain analysis of vesicular acetylcholine transporter using PET identified a distinct topographic cholinergic denervation pattern in LBD that involves specific hubs (Kanel et al., 2020). One of these hubs is the limbic network that mediates episodic memory: fornix, fimbria, hippocampus, and anterior thalamus (Kanel et al., 2020). A study using choline acetyltransferase (ChAT) immunohistochemistry, revealed the advanced loss of ChAT-immunoreactive neurons in the medial septum (Ch1) of patients suffering from dementia with Lewy bodies (Fujishiro et al., 2006). Finally, the loss of cholinergic fibers in AD and LBD could be more marked than the loss of cholinergic neurons (Hall et al., 2014; Alexandris et al., 2020). Approximately two thirds of the septal fibers to the hippocampus are cholinergic, and they are the main source of acetylcholine in the hippocampal formation (Sun et al., 2014). The septo-hippocampal cholinergic fibers are widespread in the hippocampal and they modulate the activity of the cells expressing nicotinic and muscarinic receptors (Cobb and Davies, 2005). The primary targets of cholinergic terminals are the cell bodies and proximal dendrites of the hippocampal pyramidal neurons (Frotscher and Leranth, 1985; Kiss et al., 1990). Septal cholinergic neuromodulation mediates shifting states of the neuronal excitability, providing conditions for experience-dependent increase of the synaptic connectivity (Hasselmo, 2006; Lee and Dan, 2012). The septal cholinergic neuromodulation augments the firing of hippocampal pyramidal cells in response to excitatory presynaptic activity (Cole and Nicoll, 1984). Acetylcholine is proposed to mediate the consolidation phase of memory formation (Hasselmo, 1999). It has been well-established that acetylcholine contributes to long-term memory (Woolf, 1996, 1998). Lesion studies in rodents showed that selective loss of basal forebrain cholinergic neurons causes impairment in allocentric spatial navigation (Poucet, 1989; Berger-Sweeney et al., 2001; Hamlin et al., 2013). Eliminating septal neurons by a genetic cell targeting technique to selectively eliminate cholinergic cell groups impairs spatial memory (Okada et al., 2015). Concurrently, selective elimination of nucleus basalis of Meynert affects predominantly the object recognition memory (Okada et al., 2015). This line of research demonstrates that septo-hippocampal cholinergic neuromodulation is an integral component of the hippocampus-dependent memory formation. Overall, the augmentation of cholinergic neurotransmission has been established as the first line of treatment for the episodic memory impairment of patients with AD and LBD. Patients with neurodegeneration of the basal forebrain are predominantly treated with acetylcholinesterase (AchE) inhibitors to suppress the cognitive decline and to improve the memory (Birks, 2006; Rolinski et al., 2012). Together these data indicate that dysfunctional cholinergic neuromodulation is a key component of neurodegenerative disorders with BFI and the treatment should include pharmacological agents that augment cholinergic septo-hippocampal signaling. Understanding the physiological signals that drive the activity of septal neurons would allow us to upgrade our therapeutic strategies.

## Medial Septum Mediates the Attentional Component of Spatial Memory

**FIGURE 1:**
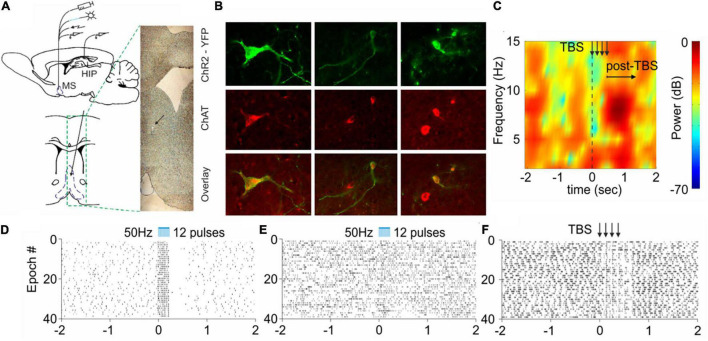
Selective cholinergic and non-selective septal stimulation effect on hippocampal activity. **(A)** Atlas schematic of experimental setup investigating the effect of septo-hippocampal stimulation (MS, medial septum; HIP, hippocampus). The histological section indicates the tip of the optic fiber and electrode. **(B)** Colocalization of ChAT staining and ChR2-YFP expression in the medial septum of ChAT:Cre rats. **(C)** Sample color-coded power spectrogram recorded in dorsal CA1 after electrical theta-burst stimulation (TBS) of the medial septum. **(D)** Raster plot from 40 repetitions of optically evoked time-locked responses of ChAT-positive cell in the medial septum. Time 0 indicates the delivery of the first train of the stimulation protocol (the scale of X-axis is in seconds). **(E)** Raster plots from of optically evoked responses from hippocampal neuron after selective optogenetic stimulation of cholinergic septal projections. **(F)** Raster plot of hippocampal cell spikes after non-selective TBS of the medial septum. The arrows show the delivery time of four trains (adapted from Mamad et al., 2015).

**FIGURE 2:**
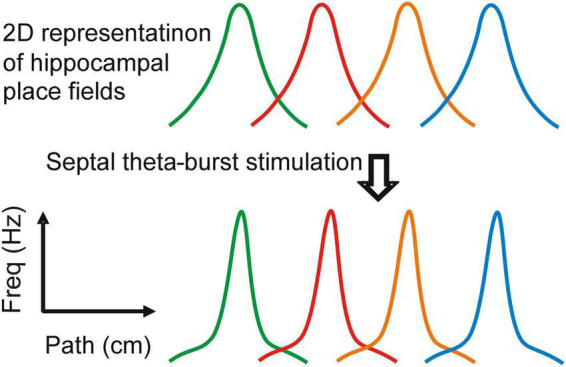
Attentional regulation of the spatial representation. Stimulation of the medial septum changes the properties of the place fields: spatial coherence, spatial information and center firing rate (based on the data from Mamad et al., 2015). The effect on the place field shape is demonstrated in two-dimentionally-represented place fields. Each place field represents the spiking of a separate place cell along linear track.

The medial septum is a region, which also contains glutamatergic (Colom et al., 2005) and GABAergic neurons (Freund and Antal, 1988). To understand better how medial septum regulates episodic memory we need to elucidate the role of the cholinergic and non-cholinergic neurons in memory formation and to identify the input signals that control the activity of these neurons. Selective activation of the cholinergic septo-hippocampal fibers enhances hippocampal function during the transition from resting to waking state (Vandecasteele et al., 2014; Mamad et al., 2015). Thus, the transition from non-active to behavioral active state is one of the main stimuli for the septal cholinergic neuromodulation. Cholinergic projections regulate the amplitude of limbic theta rhythm but their effect on hippocampal neuronal activity during active behavioral state is limited (Tsanov, 2017b). Selective cholinergic lesion of the medial septum does not affect the hippocampal theta frequency and only reduces partially theta power (Lee and Dan, 2012). Recent findings showed that the selective stimulation of GAD-positive neurons during active behavioral state entrained the spiking of hippocampal place cells (Tsanov et al., 2019) and controlled the coding properties of limbic spatially tuned neurons (Schlesiger et al., 2021). These data indicate that spiking activity of non-cholinergic neurons may have more pronounced effect during the encoding phase of memory formation compared to the cholinergic cells. If we aim to enhance the activity of septo-hippocampal projections in BFI, we should consider the stimulation of both cholinergic and non-cholinergic septal neurons. While the AchE inhibitors mediate only the cholinergic neurotransmission, an activation of all neuronal groups would be expected to have a stronger effect on the mnemonic hippocampal network. This idea is strongly supported by the finding that non-selective septal stimulation has more powerful effect on the firing of hippocampal neuronal activity, compared to cholinergic stimulation alone (Mamad et al., 2015; Figure 1). Non-selective stimulation of the medial septum had stronger effect on limbic theta rhythm compared to selective cholinergic septal activation (Mamad et al., 2015). Importantly, the non-selective septal stimulation successfully increased the center firing rate, spatial coherence and spatial information of hippocampal place cells (Mamad et al., 2015). The control of these parameters by medial septum mediates the attentional component of the hippocampal spatial representation (Figure 2). Thus, parallel activation of cholinergic and non-cholinergic septal projections to hippocampus would be expected to have stronger therapeutic effect compared to the selective acetylcholine-based treatment. As a result, the septal network effect on place cells’ spatial parameters may represent the electrophysiological substrate of spatial attention. If medial septum mediates the attentional component of spatial and episodic memory, therefore alert exploratory behavior would trigger physiologically the activity of septo-hippocampal circuitry. Next, we discuss another type of stimuli that triggers the activity of septal cells.

## Septum Neurons Integrate Reward Signals

An unexpected finding about the septal region revealed that electrical stimulation of rodent septal neurons evoked reinforcement behavioral response (Olds and Milner, 1954). The self-stimulation behavior resulting from electrical stimulation of medial septum (Olds and Milner, 1954) is likely to be mediated by the septo-hypothalamic pathway (Sinnamon, 1993). The anatomical connections to medial septum that drive reward-dependent neuronal activity arise from the hypothalamus, lateral septum and ventral tegmental area (VTA). Detailed description of how these regions provide reward signal to medial septum is reviewed in Tsanov (2017b). Lateral hypothalamus is a brain area that has been particularly implicated in feeding and reward (DiLeone et al., 2003; Harris et al., 2005; Stuber and Wise, 2016). The lateral hypothalamus is also anatomically connected to VTA and functionally dependent on the tegmental dopaminergic signaling (Shizgal et al., 1980; Bielajew et al., 2000; Nieh et al., 2013). Lateral septum is another source for reward signals to medial septum. Although the connectivity between both septal areas is still not fully elucidated, there is sufficient anatomical evidence for projections from lateral toward medial septal nuclei (Swanson and Cowan, 1977, 1979; Risold and Swanson, 1997). The third main source of reward signals comes directly from the VTA. This pathway is part of the mesolimbic dopamine system that connects ventral tegmentum with the basal forebrain and the hippocampus (Smith and Kieval, 2000). The tegmental dopaminergic projections directly innervate the neurons in medial septum (Zaborszky and Cullinan, 1996). Long-term dopamine depletion in mice is shown to attenuate the expression of choline acetyltransferase (ChAT) in the basal forebrain (Szego et al., 2011). Lesions of tegmental dopaminergic neurons result in selective impairment of basal forebrain cholinergic signaling in mice (Hu et al., 2011) and rats (Knol et al., 2014). The dopamine-dependent cholinergic impairment is particularly evident in the medial septum and dopamine deficiency may contribute to the impairment of the septo-hippocampal system (Szego et al., 2013). Septal neuronal activity related to motivational behavioral state is shown to depend on reward signals (Nishijo et al., 1997). The involvement of the septal region in reinforcement behavior is mediated directly through VTA projections (Luo et al., 2011) or indirectly through lateral hypothalamic projections (Nieh et al., 2013). It is proposed that the septal region is involved in the integration of spatial information and reward for the formation of context-dependent memory (Tsanov, 2017a). In rats performing a reward-biased reaction time task, motivational salience was encoded by the bursting response of non-cholinergic neurons in the basal forebrain that occurred before the reaction time (Avila and Lin, 2014). The authors found that faster reaction times were tightly coupled with stronger activity by the neurons in the basal forebrain in response to motivational salience signals. Artificial augmentation of the motivational salience signal in the basal forebrain via electrical stimulation resulted in faster and more precise reaction times (Avila and Lin, 2014). The neurons in the basal forebrain are involved in the motivational recruitment of attention to reward-related cues (Tashakori-Sabzevar and Ward, 2018). Reward signals to medial septum underline the neuronal involvement in motivation (David et al., 1998, 2008) and explorative behavior (Lee et al., 1988; Poucet, 1989). Finally, optogenetic activation of the septo-hippocampal GABAergic neurons in mice promoted object exploration behavior, while inhibition of the same pathway suppressed the exploration behavior (Gangadharan et al., 2016). Together, these data reveal the medial septum as a region that is being enrolled in motivational behavior. If the activity of septal neurons depends on reward signals, then pharmacological stimulation of the dopaminergic signaling could be considered as a therapeutic approach for BFI. The septal activity is engaged by behaviors that involve attention and reward. Is there another signal that could drive the activity of the neurons in the medial septum?

## Locomotor Signal is Processed by the Medial Septum

**FIGURE 3:**
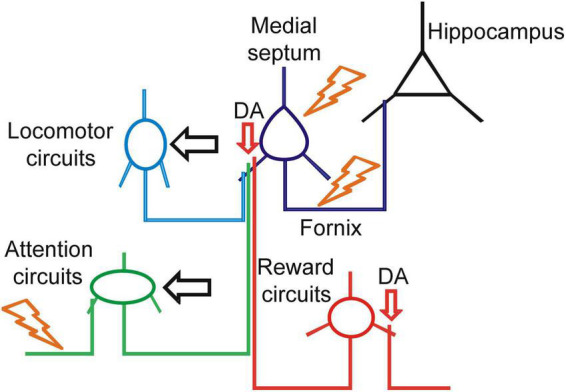
Therapeutic strategies for enhancement of septo-hippocampal signaling. Schematic representation of the translational targets for treatment of basal forebrain impairment (BFI). The orange symbols indicate electric current stimulation with deep brain stimulation (DBS) or vagus nerve stimulation (VNS). The horizontal black arrows indicate physiological activation of the circuits with behavioral motor or cognitive therapy. The vertical red arrows indicate pharmacological targets for enhancement of dopaminergic neuromodulation.

Motivation, exploration and locomotion are behaviors that generally overlap, and the septal region is proposed to be one of the brain regions where the anatomical pathways involved in these behaviors intersect (Mocellin and Mikulovic, 2021). Pharmacological inactivation or lesion of the medial septum in rodents has been shown to exert a powerful suppression of locomotion (Oddie et al., 1996). The involvement of the medial septum in the process of locomotion is reviewed in Mocellin and Mikulovic (2021). The spiking rate of the rhythmically-bursting neurons cells is significantly correlated to the animal’s running speed (King et al., 1998). Optogenetic stimulation of the glutamatergic VGluT2 cells in the medial septum induced locomotion (Fuhrmann et al., 2015). The velocity and duration of the induced locomotion were predicted by the firing rate and the number of activated VGluT2 cells. These glutamergic neurons in the medial septum specifically participated in initiation and control of locomotion activity (Fuhrmann et al., 2015). While the role of septal glutamatergic cells is directly related to locomotion the activity of the septal GABAergic neurons is also linked indirectly to linear movement. The GABAergic bursting of the cells in the medial septum is coupled to septo-hippocampal theta waves (Borhegyi et al., 2004; Bassant et al., 2005; Hangya et al., 2009). At the same time the amplitude and frequency of theta rhythm depends on locomotor speed (Whishaw and Vanderwolf, 1973; McFarland et al., 1975). One of the main correlates demonstrated for theta oscillation is the whole-body motion (Vanderwolf, 1969; Whishaw and Vanderwolf, 1973; Oddie and Bland, 1998) and theta rhythm is closely linked to the linear velocity signal (Wyble et al., 2004; Sinnamon, 2006; Geisler et al., 2007). Inactivation of medial septum disrupts path integration (Martin et al., 2007), whereas the length of traveled distance is closely linked to the parameters of theta rhythm (Gupta et al., 2012). Overall, the activity of septal glutamatergic and GABAergic neurons is associated with locomotion. If we intend to promote physiologically their spiking, we could achieve that with locomotor behavior. Next, we will discuss pharmacological, instrumental and behavioral therapeutic approaches for BFI in the context of reward, locomotor and attention signals (Figure 3).

## Dopamine-Based Treatment Strategy

It is proposed that the abolished cholinergic tone in PD dementia is closely related to the dysfunctional midbrain dopaminergic innervation of the basal forebrain cholinergic neurons (Gaykema and Zaborszky, 1996; Aarsland et al., 2021). Several studies have established the impairment of basal forebrain cholinergic function in patients with PD (Klein et al., 2010; Celebi et al., 2012; Hall et al., 2014; Bohnen et al., 2015; Muller et al., 2015). Because basal forebrain degeneration is more prominent in patients with late-stage PD, but not in the early stage, it was suggested that the dopaminergic neurodegeneration precedes the cholinergic lesion in PD (Ziegler et al., 2013). The neurodegeneration of the mesolimbic pathway is closely related to the cholinergic dysfunction across the limbic circuitry in patients with Parkinson’s disease dementia (Hall et al., 2014). It is not fully understood whether the dopamine-dependent dysfunction of septo-hippocampal network involves the direct dopaminergic projections to the medial septum, or this involves a polysynaptic pathway via the lateral hypothalamus and/or lateral septum. Regardless of the underlying mechanism there is sufficient evidence suggesting that the enhancement of mesolimbic dopaminergic signaling should be considered as one of the translational approaches for the treatment of BFI. While the dopamine-based treatment is a core element of the therapeutic plan for PD, the dopaminergic approach could be considered for other BFI disorders to treat memory impairment.

One of the most efficient therapeutic plans for patients with AD is the combination of AchE inhibitor donepezil and memantine (Howard et al., 2012; Tricco et al., 2018). Memantine is NMDA receptor antagonist, and it is part of the AD therapeutic plan because a dysfunction of glutamatergic neurotransmission is proposed to be implicated in AD pathogenesis (Bukke et al., 2020). Interestingly, memantine is known to act as an agonist at the dopamine D2 receptor with affinity that is similar to the one for the NMDA receptors (Seeman et al., 2008). Whether the dopaminergic agonism has any therapeutic role in cholinergic neurodegeneration of the basal forebrain is a question that requires further investigation. There is a small number of inconclusive studies on the effect of dopaminergic agonists on the cognition of patients with cholinergic basal forebrain dysfunction. Rotigotine is a non-selective dopaminergic agonist and it was tested as a pharmacological tool for the treatment of cognitive symptoms in AD (Koch et al., 2014). Using theta burst-stimulation protocols of repetitive transcranial stimulation (TMS) the authors demonstrated that rotigotine can restore the cortical plasticity in AD patients (Koch et al., 2014). These data complement earlier finding that dopaminergic therapy with L-DOPA, modulates cholinergic cortical excitability and leads to neurophysiological improvements in AD patients (Martorana et al., 2009). The same research group showed that rotigotine restored central cholinergic transmission of AD patients (Martorana et al., 2013). A recent study used dopamine-agonist treatment with rotigotine in patients with mild to moderate AD (Koch et al., 2020). The treatment had no effect on the Alzheimer’s Disease Assessment Scale–Cognitive subscale (ADAS-Cog) score. However, combined clinical and TMS-EEG data proposed that increasing dopaminergic neurotransmission with rotigotine enhances the cortical activity by acting on tegmental dopaminergic projections (Koch et al., 2020). A current clinical trial (NCT00306124) is investigating dopaminergic enhancement of learning and memory in healthy adults and patients with dementia and mild cognitive impairment. Further clinical trials are needed to examine the potential therapeutic effectiveness of dopaminergic drugs in patients with impaired basal forebrain. The clinical trials should consider the type of BFI, the degree of cholinergic dysfunction and the level of episodic memory impairment. The effect of dopaminergic treatment should be examined for both vascular and neurodegenerative forms of BFI. The dopamine-based therapy of memory impairment cannot be applied to all BFI disorders because some of them, including dementia with Lewy bodies, are characterized with psychotic symptoms where the dopaminergic agonism is contraindicated as a therapeutic tool. In summary, dopamine-based treatment strategy should be considered for suitable patients with acute or chronic BFI. Direct dopaminergic signal to the medial septum from VTA or indirect activation of septal afferents from lateral septum and lateral hypothalamus would enhance the function of septo-hippocampal circuitry and support hippocampus-dependent memory.

## Deep Brain Stimulation Strategy

An alternative to pharmacological therapy of BFI is the direct electrical stimulation of septal neurons or their projections. Deep brain stimulation (DBS) of the medial septum would engage not only cholinergic neurons but also the glutamatergic and GABAergic cells. DBS has been established in PD as one of the main tools in the treatment of PD and this therapeutic approach is particularly efficient in patients with pharmacologically induced motor fluctuations and dyskinesia (Kumar et al., 2000; Sankar and Lozano, 2011; Muller, 2012; Hitti et al., 2019). The anatomical target of DBS in PD is the subthalamic nucleus or internal globus pallidus, and although the stimulation of these structures has a powerful effect on the motor symptoms, DBS has little effect on the cognitive symptoms (Eisenstein et al., 2014; Merola et al., 2017). However, the effect of memory could be achieved by stimulation of the basal forebrain. A seminal study demonstrated that DBS of Ch4 (nucleus basalis of Meynert) can improve the cognitive functioning in patients with PD dementia (Freund et al., 2009). The DBS improved the memory, attention, concentration and alertness in these patients (Freund et al., 2009). Since then, DBS of the nuclei in the basal forebrain has been proposed as an innovative approach in the treatment of memory dysfunction in patients with AD and LBD. However, the attempts to establish this technique have been inconclusive. A later study failed to replicate the positive effect of DBS on patients with PD dementia (Gratwicke et al., 2018). Another study evaluated the effect of Ch4 stimulation in patients with AD where ADAS-Cog was used to assess the mnemonic performance. DBS resulted in stable or improved ADAS-Cog scores for four of the six patients (Kuhn et al., 2015a). The authors also showed that the electrical stimulation of Ch4 (Ch4-DBS) enhanced the glucose metabolism of the hippocampus and temporal lobe (Kuhn et al., 2015a). A recent study demonstrated the role of Ch4-DBS sensory gating of familiar auditory information into sensory memory in patients with AD (Durschmid et al., 2020). The therapeutic effect of DBS in the basal forebrain of patients with AD and PD dementia is reviewed by Lv et al. (2018). It has been proposed that younger patients or those at earlier stages of neurodegeneration might be more suitable for efficient DBS (Kuhn et al., 2015b; Hardenacke et al., 2016). Ch4-DBS was also tested recently in patients suffering from dementia with Lewy bodies. Although the severity of neuropsychiatric symptoms reduced in 3 out of 5 patients, there was no significant effect on the MMSE and other memory tests (Gratwicke et al., 2020). Concurrently, the authors reported functional connectivity changes after Ch4-DBS in the default mode network and the frontoparietal network (Gratwicke et al., 2020). Another study also failed to detect mnemonic cognitive performance after Ch4-DBS in patients with LBD (Maltete et al., 2021). Overall, the electrical stimulation of Ch4 (Nucleus basalis of Meynert) led to inconsistent data about the effect on cognition in patients with a dysfunctional basal forebrain (Nazmuddin et al., 2021). We could speculate that implantation of the DBS electrodes in Ch1 (medial septum) may lead to more efficient outcome particularly in the memory domain. This hypothesis is based on the findings about fornix stimulation described below. A possible explanation of the controversial findings about DBS in the basal forebrain may involve the biophysical properties of this technique. One of the main technical problems of DBS is that electrical stimulation depolarizes not only the cellular bodies of cholinergic cells, but also depolarizes simultaneously excitatory and inhibitory neurons that could in return suppress the cholinergic activity. Electrode-induced neuronal depolarization is a non-physiological process where the electric field depolarizes simultaneously different segments of the neurons, leading to antidromic spike propagation with likely involvement of distant neurons through direct axonal stimulation (Histed et al., 2009). This is one of the reasons that established optogenetics in neuroscience labs, where the physiological mode of light-induced photostimulation replaced electrical stimulation technique. While optogenetics is still far from human clinical trials due to unresolved methodological issues (Tsanov, 2020), we are still in a position to utilize the power of electrically induced depolarization. This can be done by DBS of basal forebrain but not by targeting the neurons. The targeting of their fibers can be translated in more physiological and efficient manner. The septal projections are converged in a structure known as fornix, which conveys cholinergic and non-cholinergic projections from the medial septum to hippocampus. Septo-hippocampal projections are crucial for the integrity of hippocampus-dependent memory (Tsanov and O’Mara, 2015). Fornix is described as a key anatomical element of memory formation in humans (Thomas et al., 2011). DBS of the fornix was shown to improve memory (Hamani et al., 2008). Interestingly, the authors reported that DBS of the fornix triggered feeling of *déjà vu* and recollection of autobiographical memories (Hamani et al., 2008). MMSE and ADAS-Cog tests were used in a subsequent clinical trial to examine the effect of fornix DBS on the memory of AD patients. The data revealed that fornix-DBS led to reduced decline of MMSE in 5 out of 6 patients, and improved ADAS-Cog score in 4 out of 6 patients (Laxton et al., 2010). The authors also showed augmented temporoparietal glucose metabolism and activation of the brain’s default-mode network (Laxton et al., 2010). Furthermore, 1 year of electrical stimulation of the fornix was shown to increase the hippocampal volume in in AD patients (Sankar et al., 2015). This study, which used structural MRI measurements, proposed fornix-DBS as a methodological approach for long-term structural plasticity in patients with dysfunctional septo-hippocampal processing (Sankar et al., 2015). Another study that applied fornix-DBS with the same duration of 1 year reported increased cerebral glucose metabolism in large cortical network that includes hippocampus, proposing that septal stimulation promoted the hippocampal connectivity with other brain regions (Smith et al., 2012). The authors found that the persistent metabolic increases after 1 year of fornix-DBS were greater in magnitude and more extensive in the effects on cortical circuitry compared to the effects reported for pharmacotherapy over 1 year in AD (Smith et al., 2012). The effect on cortical metabolism was confirmed in another AD study where after 1 year of fornix-DBS the mesial temporal lobe metabolism was increased and the memory scores, including MMSE and ADAS-Cog, were stabilized compared to baseline (Fontaine et al., 2013). A clinical trial also reported a therapeutic effect of fornix-DBS in AD (Lozano et al., 2016), although the findings indicated that the procedure may be more beneficial in patients aged ≥ 65 years (Lozano et al., 2016; Leoutsakos et al., 2018). The effect of septal stimulation in human studies varies and this can be explained with differences in the applied DBS parameters such as frequency, current intensity, duration and electrode impedance (Hescham et al., 2013). Dissimilar parameters, study design and sample size are the most likely reasons for the inconsistent findings (Luo et al., 2021). Establishing the most efficient protocol for DBS would be the first step of successful translation of this methodology. An ongoing clinical trial is testing fornix-DBS with personalized fornix stimulation where the DBS parameters are optimized depending on the neuropsychological scores of the patients (NCT04856072). Future steps may also include developments such as closed-loop DBS for phase-specific modulation of hippocampal activity during memorization (Senova et al., 2020). In summary, DBS of the septal neurons or their projections to hippocampus would be a suitable therapeutic approach for extensive BFI where the quality of life is considerably affected by severe anterograde amnesia. More clinical trials are needed to develop the technical parameters of this procedure.

## Vagus Nerve Stimulation Strategy

DBS is an invasive intracranial procedure, which is difficult and costly, and it is characterized with certain intraoperative risks and postsurgical adverse effects (Maslen et al., 2018). Can we stimulate the cholinergic neuromodulatory circuits without surgically invasive approach? A possible answer comes from a technique that was originally introduced to treat epilepsy. Vagus nerve stimulation (VNS) is a peripheral nerve stimulation technique that has been applied to attenuate treatment-resistant symptoms of epilepsy (Cramer et al., 2001). There are two type of VNS – invasive and non-invasive. Even the invasive type is a cheaper, easier and less risky procedure compared to DBS surgery because the electrode implantation for VNS is not intracranial, but peripheral where cervical section of the vagus nerve is surgically accessed in the carotid area of the neck (Clausen, 2010; Giordano et al., 2017). The non-invasive type of VNS involves transcutaneous vagus nerve stimulation (Hein et al., 2013; Kraus et al., 2013; Rong et al., 2016). The transcutaneous method of VNS targets the cutaneous receptive field of the auricular branch of the vagus nerve (Kraus et al., 2007, 2013; Dietrich et al., 2008; Stefan et al., 2012). Using a stimulus intensity above the sensory detection threshold, but below the pain threshold, results in a brain activation pattern similar to that of left cervical VNS (Kraus et al., 2007, 2013; Dietrich et al., 2008). VNS technique has been used in the last 20 years to control seizures in patients with epilepsy (Cramer et al., 2001). More recently VNS has been proposed as a tool for the cognitive treatment of AD (Chang et al., 2018). After 3 months of VNS treatment there was a positive effect on ADAS-Cog in 7 of 10 patients and on MMSE in 9 of 10 AD patients (Sjogren et al., 2002). Moreover, 6 months after the VNS treatment, the positive response rate was still 70% on the ADAS-Cog, and 70% on the MMSE (Sjogren et al., 2002). Based on these promising findings, the same research team recruited another seven patients with AD. The researchers followed up these total 17 patients for at least 1 year (Merrill et al., 2006). One year after VNS treatment, the score of the ADAS-Cog in seven patients stabilized or improved, while the score of the MMSE in 12 patients stabilized or improved (Merrill et al., 2006). VNS enhanced word recognition (Clark et al., 1999), improved decision making (Martin et al., 2004), improved working memory (Sun et al., 2017) and improved consolidation and memory retention in epileptic patients (Ghacibeh et al., 2006). Transcutaneous vagus nerve stimulation is shown to enhance the associative memory in older individuals (Jacobs et al., 2015). A recent study found that 6 weeks of VNS treatment triggered improvement in immediate recall and delayed recognition scores examined in epileptic patients (Mertens et al., 2022). The authors concluded that longer and more repetitive stimulation of the vagus nerve may be required to effectively modulate memory performance. Not all studies succeeded to replicate the positive effects of VNS on the cognitive performance (Dodrill and Morris, 2001; Helmstaedter et al., 2001; Hoppe et al., 2001; McGlone et al., 2008). A possible explanation about the reported variability are the different VNS parameters used in these studies as well as the different neurological conditions of the patients involved in these studies. The efficiency of instrumental methodology depends on its parameters such as stimulation frequency, duration, electrode impedance and current intensity. A current clinical trial (NCT03359902) explores the treatment of mild cognitive impairment with transcutaneous VNS. VNS might be a useful tool for the treatment of conditions with chronically impaired mnemonic processes (Vazquez-Oliver et al., 2020) but the underlying mechanisms are still under investigation. Possible hypotheses involve direct or indirect activation of the cholinergic brain network. Stimulation of the vagus nerve could directly affect the septal activity. The cells of cat basal forebrain responded to vagus stimulation with a short latency (10–15 ms) excitation-inhibition sequence (Detari et al., 1983). The authors proposed that neurons in the basal forebrain receive both excitatory and inhibitory inputs from the visceral and cutaneous receptors (Detari et al., 1983). The indirect activation of medial septum could be mediated via the aminergic reticular system. Stimulation of the cholinergic vagus nerve leads to depolarization of the neurons in its nuclei in the medulla, i.e., nucleus ambiguus and solitary nucleus, which can entrain the ascending reticular activating system (Frangos et al., 2015; Hansen, 2018). This is confirmed by electrophysiology studies demonstrating that VNS triggers noradrenergic neurotransmission (Dorr and Debonnel, 2006; Manta et al., 2009). In turn the noradrenergic reticular projections innervate the septal neurons. Noradrenergic activation of the septo-hippocampal circuitry is mediated by the projections from locus coeruleus to medial septum, diagonal band of Broca and hippocampus (Haring and Davis, 1985; Loughlin et al., 1986). Regardless of the underlying mechanism, it is very likely that the basal forebrain is one of the upstream regions engaged in the VNS signal processing and at least to some degree the VNS recruits the septo-hippocampal projections. Further research is required to elucidate the neuronal pathways involved in the VNS methodology and the efficiency of this technique in patients with BFI. In summary, VNS is a potential therapeutic tool for BFI-induced cognitive deficits. This technique can be easily applied in wide range of patients with acute and chronic BFI. We need more clinical data about the long-term efficacy of VNS.

## Locomotor Exercise-Based Strategy

For several decades aerobic exercise, including locomotion, has been shown to ameliorate cognitive dysfunction and reduce dementia risk. Exercise significantly reduced the later risks of mild cognitive impairment in number of studies (Smith et al., 2010; Ahlskog et al., 2011). A study investigating 170 participants with subjective memory impairment showed that a 6-month program of physical activity provided memory improvement measured by ADAS-Cog over a 6-, 12-, and 18-month follow-up period (Lautenschlager et al., 2008). In patients with dementia, 1 year of regular exercise resulted in significant memory improvement measured by MMSE, compared to control group of patients (Kwak et al., 2008). We know that hippocampus is an anatomical locus of exercise-induced memory improvement. Physical activity enhances human hippocampus-dependent memory (Voss et al., 2013; Duzel et al., 2016) and increases the size of the anterior hippocampus, leading to improvements in spatial memory (Erickson et al., 2011). Most of the clinical studies about exercise and cognition are correlative and they do not address the underlying mechanisms. The physiological effect of exercise on memory formation is most likely a combination of several different mechanisms including metabolic, hormonal, cellular and network mechanisms. The most common mechanism investigated in animal models is the neurotrophic effect of brain-derived neurotrophic factor (BDNF), insulin-like growth factor 1 (IGF-1) and nerve growth factor (NGF) (Adlard et al., 2004; Vaynman et al., 2004; Ding et al., 2006; Ahlskog et al., 2011; Vivar and Van Praag, 2017). The exercise-induced enhancement of memory is also associated with upregulation of hippocampal synaptic plasticity and neurogenesis (Patten et al., 2015; Vivar et al., 2016). Another way that locomotor-based exercise could promote memory formation is by physiological activation of septal neurons. Exercise is processed by the motor circuits and their activity affects the function of the limbic memory circuits. Importantly, the basal forebrain may have a designated role in this connection. The medial septum is a region that processes locomotor signals and the oscillatory and neuronal activity of septal neurons is closely related to the linear velocity speed (Tsanov, 2017b). Animal model studies have provided evidence that motor signal from the nigrostriatal network can be translated into a mnemonic signal in the septo-hippocampal circuitry. Physical activity of 12–14 weeks increases muscarinic receptor density, high-affinity choline uptake in the hippocampus (Fordyce and Farrar, 1991), as well as the number of cells expressing ChAT in some of the nuclei in the basal forebrain (Ang et al., 2006). Saporin-induced selective lesions of ChAT neurons in the basal forebrain, abolished the exercise-dependent hippocampal neurogenesis (Ho et al., 2009; Itou et al., 2011). Running exercise upregulates the input from medial septum to new neurons that correlates with augmented hippocampal neurogenesis (Sah et al., 2017). A seminal study demonstrated the effect of running on the function of the septo-hippocampal network in pyrithiamine-induced thiamine deficiency rat model of amnestic disorder. Wheel running triggered re-emergence of the cholinergic phenotype within the medial septum and diagonal band, rescued behaviorally stimulated hippocampal acetylcholine efflux and restored spatial memory (Hall and Savage, 2016). The authors presented evidence that exercise can modulate the mature cholinergic neuronal phenotype leading to improved neurotransmitter function as well as enhanced learning and memory (Hall and Savage, 2016). This line of research indicates that locomotion-based exercise promotes the physiological activation of septo-hippocampal network. Thus, locomotor exercise could be a beneficial supplementary therapeutic approach in patients with BFI. In summary, locomotor exercise should be considered as an additional strategy to aid the treatment of patients with BFI. The entrainment of septal circuitry by locomotion would provide physiological activation of septo-hippocampal circuitry that would promote hippocampus-dependent memory. Our understanding about how BFI improves memory needs further investigation of the underlying mechanisms. More studies are required to evaluate the long-term benefit of locomotor exercise in patients with acute and chronic deterioration of the basal forebrain.

## Cognitive Enhancement Strategy

Another type of exercise that could enroll the activity of septal neurons and improve their connectivity with hippocampus is not physical but cognitive. The spiking of septal cholinergic neurons is closely related to attention behavior (Tsanov, 2017b) and the septo-hippocampal signal mediates the attentional component of spatial memory (Mamad et al., 2015). Therefore, the performance of attention- and memory-related tasks would be considered as a physiological approach to recruit the basal fore brain networks and promote limbic neuronal plasticity. This type of brain exercise is known as cognitive enhancement therapy (CET). CET is proposed to be an effective therapeutic intervention for dementia (Carrion et al., 2013). A seminal multi-center, placebo-controlled study showed that CET improved cognition, behavior and quality of life in people with mild cognitive impairment or mild dementia (Han et al., 2017). MMSE and ADAS-Cog scores were significantly improved in the CET-treated group compared with mock-therapy group (Han et al., 2017). The importance of CET was demonstrated by randomized controlled trials using cognition-oriented approaches for treating people with dementia (Carrion et al., 2018). A systematic review examined CET as a therapeutic tool in patients with dementia. The authors concluded that the inconsistencies about the efficiency of CBT between different studies depend on the duration of the therapy (Carrion et al., 2018). Trials with a duration of at least 1 year showed significant effects by improving cognition and memory, while trials with a duration of less than 6 months are less likely to have significant effect on the memory scores (Carrion et al., 2018). A type of CBT that incorporates spatial components is known as reality orientation therapy. This approach uses repeatedly the presentation and information about the orientation of the patients, facilitating their comprehension about the spatial environment. While some studies showed an effect of reality orientation therapy on the memory tests such as MMSE and ADAS-Cog (Bergamaschi et al., 2013; Giuli et al., 2016), other studies failed to report cognitive improvement (Kurz et al., 2012; Alves et al., 2014; Cove et al., 2014; Orrell et al., 2017). The differences of CBT-induced effects on cognition between studies are most likely related to fact that the tested subjects had dissimilar level of neurodegeneration. Other factors for the observed variability are the testing protocols, frequency and duration of the applied CBT. The CBT clinical data mainly report correlative findings without addressing the underlying mechanisms. The mechanism behind the CBT efficiency is not fully understood, but similarly to the physical exercise, it is most likely that CBT exerts its effects via different mechanisms including metabolic, hormonal, cellular and network mechanisms. Currently, some of these mechanisms are hypothetical and more research is required for our understanding about the therapeutic effect of CBT. The most explored mechanism is the neurotrophic effect that augments neurotransmission, neurogenesis and synaptic plasticity (Mansson et al., 2016). Infusion of BDNF into the lateral ventricle of rats leads to new neurons in the septal region (Pencea et al., 2001). NGF is one of the common neurotrophic factors that regulate the survival and differentiation of neurons (Lindsay and Harmar, 1989). The NGF levels are diminished in the basal forebrain cholinergic neurons in age-related brain decline and AD (Salehi et al., 2004). On a network level CBT entrains the septal neurons that process the attentional component of memory formation (Mamad et al., 2015). In summary, sufficient clinical evidence places CBT as a strong candidate for supplementary treatment of BFI. This strategy is suitable for the majority of patients with BFI-induced anterograde amnesia. CBT is a low-cost technique, which relies on the physiological activation of septo-hippocampal pathways. We need further investigation about the cellular mechanisms and neuronal pathways that mediate the mnemonic effect of CBT.

## Discussion

The recent advances in fundamental neuroscience elucidated the function of medial septum and the information that is being processed by the septal neurons. These findings can be translated into useful treatment approaches for the therapy of patients with basal forebrain impairments. BFI involves neurological conditions that are characterized with substantial decrease of the septal neurons or impairment of their connectivity. The most common conditions that cause BFI include acute septal lesions due to AcomA rupture aneurism and chronic septal neurodegeneration due to AD or LBD. Understanding better the physiological signals that evoke the septal activity and the pre- and post-synaptic septal pathways would guide us as to type of pharmacological, instrumental or behavioral tools that can be used to enhance the septal neurotransmission (Table 1). The processing of reward signal by the septal neurons proposes a therapeutic approach with dopaminergic agents. More data are needed on how dopamine promotes septal activity in different neurological conditions characterized with BFI. Stimulation of septal activity can be conducted instrumentally by DBS methodology. Understanding better the biophysical effect of electric stimulation on the neuronal tissue would allow us to explore whether fornix DBS has a stronger effect on memory improvement compared to DBS of the septal nuclei. However, this is an invasive intracranial technique and not all patients with BFI would be suitable candidates for this procedure. Therefore, transcutaneous VNS could be used as a non-invasive approach for instrumental boost of septo-hippocampal signaling. There is still little information about how VNS acts on memory formation and which pathways are recruited by the stimulation of the vagus nerve. Knowing that septal neurons are part of the locomotor networks in the brain opens the possibility of using locomotor exercise as a supplementary therapy for BFI. Similarly, CBT is appropriately justified for the treatment of patients with a dysfunctional basal forebrain, because the septal neurons process attentional behavior. The biochemical and cellular mechanisms by which locomotor exercise and CBT engage septo-hippocampal networks should be further investigated. In summary, laboratory-based experimental advances have upgraded our understanding about how the medial septum is involved in memory formation and we should translate this knowledge into efficient treatment of neurological conditions with basal forebrain impairment.

**TABLE 1:** Summary of possible treatment strategies for basal forebrain impairment.

Treatment approach	Major positive findings	Major negative findings	Advantages	Disadvantages	Needs to be clarified
Dopaminergic agents	Koch et al. (2014): restore the cortical plasticity in AD patients Martorana et al. (2013): neuro-physiological improvements in AD patients	Koch et al. (2020): no effect on ADAS-Cog score in AD patients	Well-controlled: established as a major tool in the treatment of PD (Pahwa and Koller, 1995)	Not suitable for all BFI: dopaminergic agonism evokes psychotic symptoms in patients with advanced AD, PDD and DLB (Fernandez et al., 2003)	Efficiency, dose: clinical trials need to evaluate the effect of dopaminergic therapy on the memory formation and the optimal dose for this effect
DBS	Freund et al. (2009): improves memory and attention in PD dementia Kuhn et al. (2015a): improves ADAS-Cog in AD patients Laxton et al. (2010): reduces decline of MMSE and improves ADAS-Cog in AD patients	Gratwicke et al. (2018): no effect on MMSE in PD dementia Gratwicke et al. (2020) no effect on MMSE in DLB patients	Consistent effect: established as a reliable treatment approach in PD patients (Hitti et al., 2019)	Invasive, expensive: relies on surgical expertise, requires intracranial electrode implantation with probable adverse post-operative effects (Maslen et al., 2018)	Location, parameters: clinical trials need to evaluate the most optimal location (Ch1-4 or fornix) and the most efficient stimulation protocol including frequency and pulse duration
VNS	Sjogren et al. (2002): improves MMSE and ADAS-Cog in AD patients Merrill et al. (2006): improves or stabilizes MMSE and ADAS-Cog in AD patients Jacobs et al. (2015): improves memory in older individuals	Helmstaedter et al. (2001): no effect on memory in patients with epilepsy Hoppe et al. (2001): no effect on memory in patients with epilepsy McGlone et al. (2008): no effect on memory in patients with epilepsy	Minimally- or non-invasive: minimally-invasive VNS is established method to treat epilepsy Cramer et al. (2001), while trans-cutaneous VNS is novel anti-epilepsy treatment (Stefan et al., 2012)	Inconsistent effect: the wide variability between studies regarding the effect of VNS on memory may be due to insufficient engagement of the basal forebrain or due to the underlying pathology in the tested subjects - many of the clinical trials are conducted with patients with epilepsy (Aniwattanapong et al., 2022)	Mechanism, parameters: pre-clinical studies are needed to explore how VNS affects the septo-hippocampal circuitry Clinical trials with large cohorts need to evaluate the stimulation protocol, including frequency and pulse duration
Locomotor exercise	Lautenschlager et al. (2008): improves ADAS-Cog in patients with subjective memory impairment Kwak et al. (2008): improves MMSE in patients with dementia	Hoffmann et al. (2016): no effect on cognitive tests performance in AD patients	Low-cost: accessible and inexpensive method that could enhance the neuro-transmission and synaptic plasticity in the septo-hippocampal circuitry (Hall and Savage, 2016)	Limited effect: physiological activation of impaired brain structures could not prevent the cognitive decline in neuro-progressive degenerative impairment of the limbic system (Yu et al., 2021)	Mechanism, suitable BFI: pre-clinical studies are needed to explore how locomotion affects the septo-hippocampal circuitry Clinical trials need to establish the suitability of locomotor exercise for movement disorders
CBT	Han et al. (2017): improves MMSE and ADAS-Cog in mild cognitive impairment or mild dementia Bergamaschi et al. (2013): improves MMSE in AD patients	Orrell et al. (2017): No effect on ADAS-Cog in people with dementia	Low-cost accessible and inexpensive method that could enhance the neuro-transmission and synaptic plasticity in the septo-hippocampal circuitry (Mansson et al., 2016)	Limited effect: physiological activation of impaired limbic structures, requires consistent and continuous CBT with a duration of at least 1 year for detectable cognitive effect (Carrion et al., 2018)	Mechanism, parameters: pre-clinical studies are needed to explore how CBT affects the septo-hippocampal circuitry Clinical trials with need to evaluate the type of CBT tests and their frequency

*The main studies with positive and negative findings, the advantages and disadvantaged as well as key issues that need to be clarified for each approach in preclinical experiments and clinical trials.*

## Author Contributions

MT wrote the manuscript and approved the submitted version.

## Conflict of Interest

The author declares that the research was conducted in the absence of any commercial or financial relationships that could be construed as a potential conflict of interest.

## Publisher’s Note

All claims expressed in this article are solely those of the authors and do not necessarily represent those of their affiliated organizations, or those of the publisher, the editors and the reviewers. Any product that may be evaluated in this article, or claim that may be made by its manufacturer, is not guaranteed or endorsed by the publisher.
